# A Review of Hologram Storage and Self-Written Waveguides Formation in Photopolymer Media

**DOI:** 10.3390/polym9080337

**Published:** 2017-08-03

**Authors:** Ra’ed Malallah, Haoyu Li, Damien P. Kelly, John J. Healy, John T. Sheridan

**Affiliations:** 1 School of Electrical and Electronic Engineering, UCD Communications and Optoelectronic Research Centre, University College Dublin, Belfield, Dublin 4, Ireland; raed.malallah@ucdconnect.ie (R.M.); damienpkelly@gmail.com (D.P.K.); john.healy@ucd.ie (J.J.H.); 2 Physics Department, Faculty of Science, University of Basrah, Garmat Ali, Basrah, Iraq; 3 Department of Biomedical Engineering, Stony Brook University, State University of New York, Stony Brook, NY 11794, USA; haoyu.li@stonybrook.edu

**Keywords:** photopolymer, holography, self-written waveguide

## Abstract

Photopolymer materials have received a great deal of attention because they are inexpensive, self-processing materials that are extremely versatile, offering many advantages over more traditional materials. To achieve their full potential, there is significant value in understanding the photophysical and photochemical processes taking place within such materials. This paper includes a brief review of recent attempts to more fully understand what is needed to optimize the performance of photopolymer materials for Holographic Data Storage (HDS) and Self-Written Waveguides (SWWs) applications. Specifically, we aim to discuss the evolution of our understanding of what takes place inside these materials and what happens during photopolymerization process, with the objective of further improving the performance of such materials. Starting with a review of the photosensitizer absorptivity, a dye model combining the associated electromagnetics and photochemical kinetics is presented. Thereafter, the optimization of photopolymer materials for HDS and SWWs applications is reviewed. It is clear that many promising materials are being developed for the next generation optical applications media.

## 1. Introduction

In recent years, photopolymer materials are being actively studied for practical applications such as Holographic Data Storage (HDS) [1,2,3,4,5,6,7], hybrid optoelectronics [8,9], and Self-Written Waveguides (SWWs) fabrication [10,11,12,13,14,15]. Their versatility, flexibility, permanent (stable) nature, ease of use, and self-processing character provide many advantages over more traditional passive photosensitive materials such as silver halide (AgHa), dichromated gelatin (DCG), and photosensitive glass [16,17,18]. In this paper, we report on our study of the behavior of photopolymers for volume Holography and SWWs applications. We have three main aims, all of which involve novel research: (i) To increase our understanding of the physical electromagnetics and photochemical kinetics, taking place within photopolymer materials during the holographic grating exposure and SWWs formations. (ii) To improve both the accuracy and flexibility of a three-dimensional (3D) Nonlocal Photo-polymerization Driven Diffusion (NPDD) model, which can be used to describe in greater generality the time varying photophysical and photochemical evolutions taking place in the holographic grating depth during exposure. This full 3-D model will enable a more accurate physical description of the evolution of the refractive index modulation during holographic grating formation in material layers. (iii) To investigate and explore, both numerically and experimentally, the formation of self-written waveguide structures in dry thick Acrylamide Polyvinyl Alcohol (AA/PVA) based photopolymer samples. The nonlinear photo-absorptive effects taking place during the photo-initiation processes are included in the theoretical model. In this way, the optically induced growth of SWWs in such a free radical photo-polymerization systems and the corresponding temporal and spatial light intensity distribution within the materials can be examined. We discuss in detail how the NPDD model can be used in fiber optic applications.

In this paper, we begin by introducing photopolymer materials in general and discuss the photopolymer material (Acrylamide/Polyvinyl Alcohol AA/PVA) used throughout this article. We give a description of the preparation of the AA/PVA photopolymer. In the second section, a brief review of the fundamental theory of holography, optical diffraction by thin and thick (volume) gratings [19], and a short description of the recording of unslanted transmission type volume holograms are presented. Then a brief synopsis of Kogelnik’s first-order two-harmonic Coupled Wave Theory (CWT) of volume holographic diffraction [20] is presented. In the third section, we examine the self-writing mechanism providing a detailed theoretical account and reviewing some of the reliant background literature reviews of different materials are discussed with several resulting applications.

## 2. Photopolymers

Photopolymers are light sensitive polymeric materials, which change their physical and/or chemical properties when exposed to light. Photopolymers are used in many different optical systems and for many different purposes. For example, they can serve as the recording medium in holography or can be used to produce SWW, as discussed in this paper. In this section, a general review of photopolymers and some application related information is presented.

### 2.1. Photopolymer Materials Modeling and Applications

In Close et al. (1969), the use of photopolymer material was first examined as a holographic recording material and its various uses and productivity [21]. Only a small number of commercially viable holographic recording systems using photopolymer material have been produced, after numerous material systems were examined and researched [22,23]. Polymer materials are of interest because they have several practical advantages. Thick layers can be fabricated so the resulting gratings can act as Volume Holographic Gratings (VHG), giving high diffraction efficiency with good angular selectivity. The self-developing of most materials require only some simple post-processing steps, for example an exposure to light or heat treatment. Therefore, this will eliminate the need for wet chemical development, because these photopolymers are suitable for holographic and waveguides applications [24,25,26,27], and also holographic data storage [22,28,29,30,31,32,33,34,35,36,37,38].

In general, the photopolymers consist of a monomer, a photosensitive dye and an initiator, which can either be as liquid or dry layer systems. Dry photopolymer layers usually contain a polymeric binder in addition to the other components. This liquid material consists of a mixture of acrylamide and metal acrylate monomers and a photo-catalyst methylene blue, held in place between two glass plates for recording. Diffraction efficiencies as high as 45% were achieved with exposures of around 30 mJ/cm^2^. The resolution of the photopolymer was found to be around 1000 lines/mm, however the material had a very short shelf life. The media’s sensitivity are studied with the aim of decreasing the necessary exposure (0.6 mJ/cm^2^) using lead or barium acrylate along with acrylamide as the monomer; these improvement discussed by Jenney et al. [39,40].

A second liquid system was developed by Sugawara and Sukegawa [41,42]. It consisted of an acrylamide monomer, a cross-linker (*N*,*N* methylene bisacrylamide), sensitizing dye (methylene blue) and either triethanolamine (TEA) or acetylacetone as an initiator. Diffraction efficiencies of 65% were obtained with an exposure energy of 50 mJ/cm^2^ and a resolution of 550 lines/mm. Using a ferric ammonium citrate as sensitizer and *t*-butyl hydrogen peroxide, the material based on the same monomer and cross-linker was examined [42]. It gives 80% diffraction efficiencies during exposure to 20 mJ/cm^2^ with a resolution of 1500 lines/mm.

Sadlej and Smolinska [43] improved the original system proposed by Close et al. [21] by including a poly-vinylalcohol (PVA) binder. This allows the production of dry photopolymer layers that are superior to liquid systems. In this way, the shelf-life of the original material was greatly improved, demonstrating conclusively that a binder can improve the stability of recorded holograms. The main disadvantage was the low diffraction efficiency which was less than 4%.

Jeudy and Robillard [44] reported an interesting version of the liquid Sugawara system, which included a reversible photo-chrome (indolino-spiropyran), as sensitizer and PVA as binder. The photo-chrome, which was fully transparent in visible light, could be activated by ultra-violet (UV) light shifting its absorption band to allow recording at 633 nm. After completing the recording (switched off the UV), the photo-chrome was rendered inactive, resulting in a highly transparent stable hologram of typically 90% diffraction efficiency and an exposure energy of 100 mJ/cm^2^ with a resolution of 3000 lines/mm.

In 1987, Calixto [45] presented more works on acrylamide-based systems. Such as the material contained acrylamide monomer, TEA as an electron donor, methylene blue photosensitizer and PVA as a binder. The exposure energy was low, i.e., approximately 94 mJ/cm^2^, and the maximum diffraction efficiencies reported were around 10%.

By the early 1990s, Fimia et al. [46,47], had devised a method for increasing the sensitivity of acrylamide photopolymers by reducing the inhibition time caused by oxygen. The material contained two dyes: (i) methylene blue; and (ii) Rose Bengal, sensitive to 633 nm and 546 nm, respectively. The layer was pre-exposed to a 546 nm beam before actual recording. The Rose Bengal dye produces radicals that be reacted with oxygen within the layer. There is a reduced amount of oxygen and sufficient methylene blue dye to polymerize the monomer, which takes place after recording at 633 nm. This system has a diffraction efficiency of 40% at a spatial frequency of 1000 lines/mm which could be obtained with an exposure of 3 mJ/cm^2^.

The Calixto photopolymer was sensitized by Martin et al. [48] for recording in the 514 nm region by the addition of a xanthene dye. Diffraction efficiencies of greater than 80% were obtained with an exposure energy of 80 mJ/cm^2^. The spatial frequency of the material was approximately limited to below 2750 lines/mm.

Weiss et al. [49] improved the sensitivity of the Close and Sugawara material at 514 nm by adding diphenyl iodonium chloride to act as a sensitizer along with TEA. The addition of glutaraldehyde as a second cross-linker was found to increase the refractive index modulation. Diffraction efficiencies of greater than 90% at 2000 lines/mm were shown to be achievable with an exposure of 12 mJ/cm^2^.

Blaya et al. [50] improved the sensitivity of the acrylamide material for recording at 633 nm by changing the cross-linker used. This new cross-linker, *N,N*-dihydroethylenebisacrylamide, caused an increase in the rate of polymerization which lowers the exposure energy required. Diffraction efficiencies of 70% at a spatial frequency of 1000 lines/mm have been obtained with an exposure of 5 mJ/cm^2^.

In 1998, Blaya et al. [51] investigated the effects of the addition of a cross-linking agent (*N*,*N*′-methylenebisacrylamide) to a photopolymerizable matrix for HDS. They observed a nonlinear response of the material with regard to the storage intensity. A diffraction efficiency of around 88% was achieved with an energy exposure of 12 mJ/cm^2^. At that time, the sensitivity of this material approached that of commercial films without the use of post-processing, which was necessary in other materials.

Later in 1998, Blaya et al. [52] examined the effects of exposing intensity, thickness, and variation of the concentration of each component. Diffraction efficiencies of 80% and energetic sensitivities of 40 mJ/cm^2^, were obtained in photosensitive films of a 35 μm thickness at a spatial frequency of 1000 lines/mm.

A hybrid material containing acrylamide and acrylic acid as monomers was proposed by Zhao et al. [53]. Materials such as methylene blue, TEA and *p*-toluenesulfonic acid and gelatin are used as photosensitizer, sensitizers and binder, respectively. They can record up to 4000 lines/mm. The maximum diffraction efficiency obtainable was over 80% and the exposure energy required was 2 mJ/cm^2^.

In 1999, Mallavia et al. [54] developed a photopolymerizable system composed of a xanthene dye, *N*-methyldiethanolamine (MDEA) as photoinitiator, with a monomer mixture of 2-hydroxyethyl metacrylate (HEMA) and pentaerithritol triacrylate (PETA). The best results reported were diffraction efficiencies at over 40% with energy exposures of 800 mJ/cm^2^, with a decrease of inhibition time when high concentrations of PETA (>67%) were used.

Meanwhile, Fimia et al. [55] reported a new aqueous photopolymer containing the monomers acrylamide, *N*,*N*′-methylenbisacrylamide and zinc acrylate, the initiators 4,s-diiodosuccinylfluorescein (2ISF) and methylene blue (MB), and the co-initiator sodium *p*-toluenesulphinate. The sensitivity of high energy, at 514 nm or 633 nm, was compared to that of the same mixture but with only one of the two dyes. The combined action of the cationic and anionic dyes as visible photo-initiators, lead to maximum diffraction efficiencies of 15–20% for 15–60 mJ/cm^2^. It is worth mentioning form these enhancement that suggested as such mixture could be of potential utility when recording multifunctional diffractive systems, which arose due to the developer mixture in photographic emulsions.

In 2001, O’Neill et al. [56] examined the use of an aerosol sealant (artist varnish) to seal the photopolymer holographic recording material from the environment. Application of the sealant was found to create some deterioration in the optical quality of the resulting holographic optical elements (HOE). However, it was also shown to produce an increased lifetime of the active material (pre-recording); in addition, an increase of the diffraction efficiency from the resulting diffraction gratings recorded as well as an improved HOE shelf-life. While some of the material characteristics were improved by the introduction of the varnish layer, there was an increase in diffuse scattering by the recording layers. This technique was studied in order to replace cover-plating as it was found to be a cheaper and simpler way for sealing the photopolymer recording material from the environment. In general, the cover-plating of material layers will be lead to get a better humidity stability, reduced inhibition effects and more stable long-term diffraction efficiency [46,47].

In 2002, in the work reported by Yao et al. [57], an acrylamide-based polymeric film was optimized and characterized for holographic recording. Diffraction efficiencies of 55%, with an energy sensitivity of 60 mJ/cm^2^, were obtained in the photosensitive films of 150 μm thickness with a spatial frequency of 2750 lines/mm. The article indicated that each component had an optimal concentration, i.e., it is not the case that the higher the concentration, the better the holographic recording characteristics.

In the same year, Suzuki et al. [58] demonstrated a stable holographic storage solution using green light to expose the hologram. The resulting holograms have high diffraction efficiencies and recording sensitivity due to the addition of TiO_2_ nanoparticles which were dispersed in the methacrylate photopolymer films, has been studied. It was shown that the diffraction efficiency, as well as the recording sensitivity, significantly increases as the nanoparticle concentration is increased. It was also found that volumetric shrinkage during holographic exposure is noticeably suppressed by their inclusion. A fractional change of 2.9% in thickness was estimated.

In 2003, an improvement of more than one order of magnitude in the holographic recording sensitivities in the green using pyrromethene dyes with SiO_2_ nanoparticle-dispersed methacrylate photopolymer films, was reported by Tomita and Nishibiraki [59]. This work extended the results presented in [58]. Using several dye concentrations and writing intensities, the holographic recording dynamics were measured. An optimum recording intensity for a given dye concentration to maximize the strength of a permanent (fixed) volume hologram was also measured. It was also shown that a dye concentration lower than 0.1 wt % was preferable when the thickness of the sample layer was 50 μm. The sample with 0.05 wt % of dye exhibited increased sensitivity *S* and dynamic sensitivity S^*^ by factors of 9 and 12 times the values, of 4.2 × 10^−4^ and 1.2 × 10^−3^ cm^2^/J, for the corresponding undoped samples.

Later in the same year, Blaya et al. [60] reported a photopolymerizable dry polymeric film based on pyrromethene dye. This new composition contained three components: poly-(methylmethacrylate) as a binder, 2-hydroxyethylmethacrylate as a monomer and 1,3,5,7,8-pentamethyl-2,6-diethylpyrromethene-difluoroborate as photo-initiator. After using the thick layers (500 μm), diffraction efficiencies close to 60% and exposures of ≈1 J/cm^2^ can be obtained. When the concentration of monomer and the exposing intensity were changed, the properties of the material were also examined. This system shows good results for low exposure intensities, i.e., this material could be used for low power applications.

It is important to see the development of these materials in the context of contemporary process in applications. In 2005, McLeod et al. [33] demonstrated that the addition of a reflecting head and a confocal pinhole for homogeneous holographic photopolymer disk lead to a standard optical drive that can read and write many layers of micro-holographic tracks in a rapidly rotating. The analysis presented revealed that the index change available for each layer is the total possible change over the number of layers, analogous to results for page-based holographic multiplexing. Experimental studies have shown that photopolymer materials have been used extensively in the holographic storage field, e.g., used in a modified traditional optical drive to achieve performance comparable with page-based holographic storage but with a reduced write/read system complexity. What distinguishes the page-based holographic storage is that the rapid-disk rotation of the bit-based storage permits faster access time, therefore the total addressable storage space was shown to be as large as page-based systems at comparable operating points. The analysis was experimentally validated at 532 nm during write/read 12 layers of micro-holograms using 125 μm photopolymer disk continuously rotating at 3600 rpm. Using a system consisting of wavelength and numerical aperture of commercial Blu-Ray systems, can be predicted a capacity limit of 140 Gbytes in a millimeter-thick disk or over 1 Tbyte. It is also important when discussing material development to be aware of developments in the modeling and characterization of such materials.

Kelly et al. [61] proposed an extension to the 1-D Nonlocal Photopolymerization Driven Diffusion (NPDD) model [62,63] to account for the temporal response associated with polymer chain growth in AA/PVA material. The response of exponential temporal material along with the non-local spatial material response function was achieved. It should be noted that previously been assumed all temporal effects were instantaneous, and were thus neglected. Using rigorous coupled wave analysis (RCWA) (i.e., electromagnetic simulations of volume holographic gratings) [64] and the Lorentz–Lorenz expression (to describe the material index), the temporal development of refractive index modulation was determined during and after illumination. Then, the comparisons between the theory and experiment for two model were made. Initially, the mechanism of dominant termination was assumed to be bimolecular (two long chains), then assumed that the primary termination (one radical) to be dominant. For both models, the material parameters has been extracted which is based on the best fits with experimental data. These fitting were achieved by assuming the primary termination model in higher correlation.

O’Neill et al. [65] examined the temporal evolution of an optically induced surface relief pattern on the surface of AA/PVA based holographic recording materials layers during and after exposure to intense light. They examined the effects of coherent single and two beams exposure energy, i.e., the final surface relief pattern formed. The temporal evolution of the resulting surface profile was monitored. A 24 mJ/cm^2^ beam was used to expose a 44 μm thick photopolymer layer. An initial shrinkage (~0.34 μm) was clearly shown, followed by a swelling period. Then, the resulting profile was a pedestal ~1.2 μm in height. In addition, they examined the effects of varying the exposure energy on the resulting temporal behavior of the central height. The difference between the edge height and that at the center of the exposure, is known as a height parameter. As expected, the material variation was increased during increases of intensity. During the examination of the effects of exposing the layer with both a single beam and a double beam illumination, it was found that although the swelling is less for the two recording beams which is still appreciable. Both 47- and 50-μm-thick layers were examined using the double beam and single beam exposures, respectively. In both cases, the exposure energy was ~240 mJ/cm^2^. It is worth mentioning that the holographic recording as shrinkage/swelling of the material can be affected by these results, and thus can lead to Bragg detuning effects. In particular, this is an important for slanted gratings. In addition, this study has enabled the possibility for using the volume change in holographic recording materials to produce patterned surfaces.

The decreasing of the extent of the nonlocal effects within a material will improve its high spatial frequency response; this is one of the key predictions of the 1-D NPDD model [62,63]. In 2008, Gleeson et al. [66] demonstrated that the development of the spatial frequency response of an Acrylamide/Polyvinyl Alcohol based photopolymer was reported through the addition of a Chain Transfer Agent (CTA), sodium formate. The nonlocal response parameter was reduced through the CTA effects on the decreasing of average length of the polyacrylamide (PA), σ while maintaining a high rate of polymerization. Thus, the chain transfer kinetic effects introduced by the CTA, which contribute to the increased localization of the polymerization, indicated an increase in the concentration of the monomer radicals available for bimolecular termination, and also an efficiency of less than 100% in the re-initiation process.

In addition, an algorithm for determining the appropriate HDS recording schedule based on the physical properties of the AA/PVA recording medium, using a more rigorous 1-D NPDD formulation, is proposed by Kelly et al. [67]. The algorithm’s predictions with inverse-square scaling law of holographic diffraction, for various multiplexing schemes, were examined experimentally. A significant break down of low numbers for high diffraction efficiency gratings, was shown using the scaling law. It was shown that the experimental results fitted quit well with the numerical predictions from the theoretical model. The largest discrepancy in all cases occurred for the final exposure, where the reduced monomer concentration available, and possible grating non-uniformities, strongly affect the required exposure schedule time. Using Kogelnik’s model, the study explained the scaling relationship between the diffraction efficiency of each grating and the numbers of gratings recorded, for multiple high diffraction efficiency gratings.

In the work presented in 2010 by Gleeson et al. [68], the 1-D NPDD model applied to AA/PVA systems to more accurately model the effects was developed, include: (i) time varying primary radical production; (ii) the rate of removal of photosensitizer; and (iii) inhibition. Thus, the spatial and temporal variations were included into the primary radical generation for the first time. Therefore, the extensions of the NPDD model provide a more physically intuitive representation of the processes that occur during free radical photo-polymerization. In addition, the effect of oxygen diffusion from outside the material layer was also incorporated in this model by including a rate of oxygen replenishment from the surrounding environment, thus allowing for more accurate modeling of the inhibition, which can dominate the initial period of exposure.

Inhibition effects are particular important at the start of exposures and for low energy (large area) exposures. Dye performance is critical throughout the exposure process. Guo et al. [69] examined the effects of photosensitizer diffusion “Erythrosine B (EB)” in AA/PVA material. These have been achieved using simple experimental techniques and the use of a diffusion model. Diffusion rate of EB was estimated by *D* ≈ 6.27 × 10^−^^12^ cm^2^/s. In such photopolymer layers, the rate of diffusion of the AA monomer has been reported to be in somewhere the range of 10^−^^11^–10^−^^10^ cm^2^/s [70,71,72]. The molecular weights of EB and AA are 879.86 g/mol and 71.08 g/mol respectively. However, the molecular weight alone does not determine the rate of diffusion. Therefore, it would be reasonable to expect EB to diffuse more slowly than AA. The results show that EB diffuses at least an order of magnitude slower than AA monomer in the same layers.

Then, Sabol et al. [73] introduced a detailed study on the photo-initiation process of the photosensitizer (Irgacure 784) used in a photopolymer which composed of an epoxy resin matrix and vinyl monomers [74]. A precursor of the inphase photopolymer. Irgacure 784 is a type of titanocene photo-initiator, which has the property that it does not require a co-initiator to produce the free radical. Sabol et al. has thoroughly discussed the photochemical reactions Irgacure 784 involved. Importantly, the experimental results reported verify that multiple different absorbers, i.e., excited states of the dye, are simultaneously present in the layer at different stages during the exposure.

Later in 2011, the work demonstrated by Gleeson et al. [75], developed analysis tools for 1-D NPDD based modeling of the mechanisms which occur in AA/PVA photopolymers during and post-exposure that can be used to predict the behavior of many distinct types of material for a wide range of recording conditions. In this work, further extending the NPDD model was reported to clearly quantify some of the trends the model predicts. Thus, their implications for attempts to improve photopolymer material performance can be analyzed. During these attempts of optimizing the performance of a photopolymer material, the results obtained are of high practical importance. As the different types of monomer have diverse chemical and structural characteristics, the knowledge of these characteristics is necessary. One of the implications of the predictions of this model is that producing a higher refractive index modulation, which occurs by using a monomer with a large propagation rate constant and low bimolecular termination rate will produce a higher refractive index modulation. To increase the dynamic range of the photopolymer and to maximize the index modulation achievable, it is also desirable to have a monomer with high mobility (a fast diffusion rate). It is also worth noting in this work that the optimum refractive index modulation cannot be obtained, this occurs when the bimolecular termination rate is too small or the propagation rate is too large. Thus, deleterious effects are compounded further when increased the material viscosity as a result of polymerization.

Liu et al. [76] revised the NPDD model in order to make it suitable to characterize the behavior of the PQ/PMMA photopolymer material. Based on a detailed analysis of the photochemical and material transport mechanisms present in PQ/PMMA photopolymer during holographic grating formation, a set of NPDD rate equations are derived, governing the temporal and spatial variations of each associated chemical species concentration. As noted, the model fits the experimental results satisfactorily.

The results of a new acrylate based photopolymer material developed by Bayer Material Science (BMS) [77], were reported by Gleeson et al. [23]. This material was examined by various optical techniques and then characterized using the NPDD model. When estimating a very low nonlocal parameter value of 9.2 nm, the refractive index was modulated up to Δ*n*_sat_ = 8 × 10^−3^. Through the comparison of the materials AA/PVA and BMS, it found that the BMS material has several features, including: (i) a substantially faster response of the refractive index modulation which respect to the recording dosage especially at lower power densities; (ii) the ability to achieve three times higher refractive index modulation; (iii) a six times smaller nonlocal response parameter, which means a little drop-off in material response up to 5000 lines/mm; and (iv) at high spatial frequencies, that performance of high diffraction efficiency holograms recorded was improved. In this work, the capabilities of a new class of photopolymer has been demonstrated. Thus, they can be manufactured on an industrial scale as large area plastic films, full color recording, offering high index modulation, high light sensitivity and environmental stability.

Earlier, the NPDD model was extended [66] to include the kinetics of chain transfer and re-initiation to analyze the effects of various Chain Transfer Agents (CTA) on the system kinetics and to study their use in reducing the average polymer chain length in free-radical based photopolymer materials.

Following on from this, Guo et al. [78,79] reported a study for AA/PVA based photopolymer material containing chain transfer agents. A comparative study has been also reported for the effects of two different types of chain transfer agent: Sodium Formate (HCOONa) and 1-Mercapto-2-Propanol (CH_3_CH(OH)CH_2_SH). The validity of the proposed NPDD model was examined and matched with experimental data [78,79]. Thus, it confirmed that the average length of the polymer chain was formed by adding the chain transfer agents, as is the resulting nonlocal response of the materials. The most effective material combination used resulted in decrease of the nonlocal parameter, from 61 nm to 41 nm. It is worth noting that the comparing the two types of transfer agents shows that the results achieved by 1-mercapto-2-propanol, were better than those using sodium formate. CTAs was produced an improvements in the high spatial frequency material response, reaching up to ~28% increase in the refractive index modulation which achieved at 3000 lines/mm.

In 2012, Cody et al. [80] demonstrated the development of a new non-toxic holographic photopolymer material. The photopolymer composition has been optimized for the non-toxic monomer, and its spatial frequency dependence on the refractive index modulation in the transmission mode of recording has been investigated. The new material has been reported to be capable of producing refractive index modulations of up to 3.3 × 10^−3^, resulting in diffraction efficiencies >90% in 70 µm thick layers. It is suitable as an environmentally friendly alternative to the known acrylamide photopolymers for holographic applications. However, further characterization of the material’s recording capabilities including its spatial frequency response is necessary.

Ortuño et al. [81] reported work with a photopolymer, biophotopol, with higher environmental compatibility than acrylamide based photopolymers. Ortuño et al. observed good performance in 900 μm thick layers. Diffraction efficiencies, DE_max_ = 77% for exposures of 200 mJ/cm^2^ were obtained. Biophotopol appears potentially useful for recording many holograms, i.e., by multiplexing a small angular interval of the angular response curve (0.2°). In this photopolymer, biophotopol, a sodium salt 5′-riboflavin monophosphate (PRF) was used as a dye. Taking into consideration that this material is soluble in water and found in the environment, it is unlikely to cause environmental problems. Additionally, the by-products derived from PRF after photodecomposition are also dyed which can be initiated a new polymerization reactions with a similar efficiency to that of the initial PRF molecule. Therefore, dye bleaching does not seem to be a limiting factor when several holograms are recorded, which is the opposite evaluation of what happens in the case of many photopolymers in the by-products of the reaction of Eosin Yellowish (EY) and Triethanolamine (TEA), i.e., they are colorless. As with each new hologram recording the dye concentration is decreased, the decreasing dye concentration is a limiting factor, resulting in a decreasing sensitivity of the polymer layer for each holographic recording. Furthermore, Ortuño et al. reported the main parameters related to the kinetics of the photopolymerization reaction. This photopolymer appears to offer a potentially useful alternative for use in producing holographic optical elements and for data storage. More recently, Navarro-Fuster et al. [82] have shown a method to improve the energetic sensitivity of the biophotopol material by tuning the recording wavelength from 514 to 488 nm. It has been demonstrated that the diffraction efficiencies of 90% at a spatial frequency of 1205 line/mm were obtained in 300-μm-thick layers with exposures of 300 mJ/cm^2^.

In 2013, Sabel et al. [83] demonstrated the investigation of kinetics of volume hologram, which forms in an organic cationic ring-opening polymerization system. However, the potential impact of living polymerization is also studied. For this material of the grating formation process positive change of the refractive index contrast on a molecular level and a negative contribution due to component segregation (mass transports). These two act as competing effects, taking place simultaneously but over different time scales resulting in two-step growth curves. The measured quantity used to optically determine the modulation only depends on the absolute value of the modulation. The sign of the modulation (grating position) becomes particularly important with regard to integrated optics. The intrinsically negative refractive index contrast notwithstanding, waveguide functions are still feasible.

The influences of the varying functionalities of types of thiols used as chain transfer agents on the material spatial frequency response, shrinkage and thermal stability, were reported by Guo et al. [84]. This study involved the examination of a volume grating recorded in a photopolymerizable composite material film ZrO_2_ nanoparticles containing dispersed Nanoparticle-Polymer Composites (NPCs). It is demonstrated that the incorporation of multifunctional thiols results in: (a) a substantive increase in Δ*n*_sat_ at spatial frequencies as high as 3333 lines/mm; (b) much lower shrinkage; and (c) a reduction in thermal changes compared to mono-thiol doped NPC volume gratings. It can be shown that the incorporation of tri-thiol gives the best overall performance from amongst the three thiols examined. These results are important for understanding the role of multifunctional thiols on the holographic recording properties of photopolymer and NPC materials.

In 2015, Guo et al. [85] reported the results of an experimental investigation of the properties of NPCs doped with chain transferring multifunctional di- and tri-thiols as chain transfer agents. Guo et al. identified the optimum concentrations for mono- and multifunctional thiol-doped NPC film, at which the largest values of Δ*n*_sat_ are obtained, and compared the results to those of undoped NPC sample. It is shown that their incorporation into NPCs more strongly influences recording than mono-thiol doping. As in the case of mono-thiol doping, there exist optimum concentrations of di- and tri-thiols that maximize the saturated refractive index modulation. It is also shown that the recording sensitivity monotonically decreases as the multifunctional thiol concentration increases due to the partial inhibition of the photopolymerization event due to the presence of excessive thiols.

Kawana et al. [86] have proposed a spectral interferometric technique for measuring the evolution shrinkage in a photopolymer material. The proposed method is simple and not strongly effected by environmental disturbances since a single probe beam is employed. It provides information on the overall shrinkage evolution of a photopolymer material during curing. They have applied it to examine blue-sensitized NPC material. A discrepancy in steady-state shrinkage between the spectral interferometric and holographic Bragg-angle detuning measurements is also explained.

In 2016, Shelkovnikov et al. [87] studied the sensitivity of the optimized photopolymer material (sensitized by thio-erythrosin). It was shown that these values are 180 mJ·cm^−2^ at 633 nm, 480 mJ·cm^−2^ at 658 nm, and 1.3–1.7 J·cm^−2^ at 690 nm. It is worth noting that the sensitivity of conventional photopolymer materials while recording at the permitted transitions is just a slightly stronger than the sensitivity of this photopolymer material. However, the material has one or two orders less absorption, allowing holographic recording without the usual distortions caused by strong absorption. Using the wavelengths 633, 658 and 690 nm, the evaluation for the absorption of photopolymer used is comparable with scattering by typical inhomogeneities within a photopolymer matrix which is not related to absorption of the dye. The photopolymer material used is sensitive to the red region of the spectrum to the singlet-triplet absorption of the dye-sensitizer. Thioerythrosin triethylammonium was compared with the dyes studied (xanthene and thioxanthene), which were identified as the most effective dyes. Results from studying the holographic recording with the use of forbidden singlet-triplet electronic transitions of the dye-sensitizer in the photopolymer were reported. It is worth noting that the absorption spectral region of the main transition of the dye is in the green, and also the absorption spectral region of the singlet-triplet transition is in the red, up to 690 nm. Furthermore, in this paper, a better solvent was identified for this photopolymer composition. Replacement of chloroform with acetone permitted an increase the photopolymer sensitivity by factor of more than three. A detailed search for the optimal concentrations of the key material components allowed a further increase in the sensitivity of the photopolymer by factor of 1.6. Low photopolymer absorption permitted seven multiplexed reflection holograms to be recorded.

Clearly, there has been an extensive amount of work done in the area of photopolymer material modeling in recent years. A full understanding of the photophysical and photochemical processes, taking place within photopolymer materials, is of great importance in order to develop a fully comprehensive model, which includes all major photophysical and photochemical effects.

The material used in this study is essentially based on the Calixto photopolymer system [45]. It is an Acrlyamide/Polyvinyl Alcohol (AA/PVA) photopolymer, which contains a monomer (acrylamide), a crosslinker (bisacrylamide), electron donor (triethanolamine, TEA), xanthene/thiazine activating dye, and a binder (polyvinylalcohol, PVA). Its material composition and preparation are described in the next subsection.

### 2.2. Material Composition; and Preparation of the Hologram Layer and SWW Bulk

**Figure 1:**
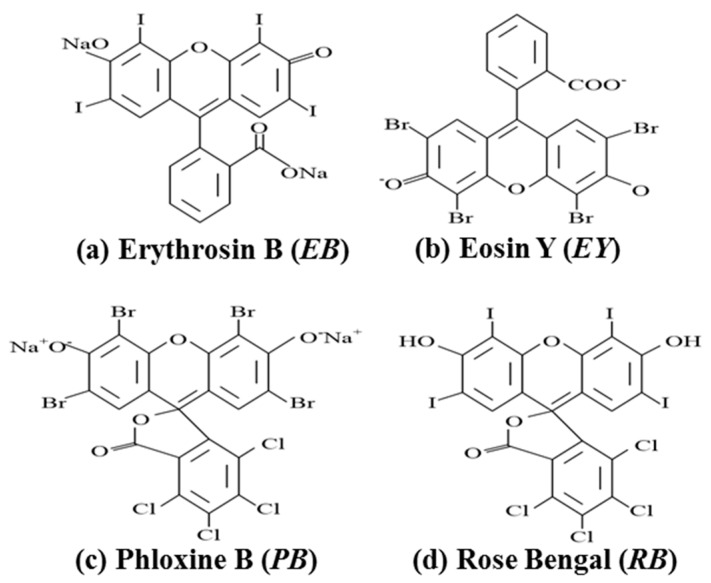
Structural formulas for four different photosensitizers.

Photopolymer materials can be made sensitive to a particular wavelength by choosing a photosensitizing dye. If a Xanthene dye is used the holographic recording can be carried out using a green, e.g., λ = 532 nm laser. The dyes examined absorb light and act as a photo-initiator of the polymerization process. Figure 1 shows the different structural formulas of the four Xanthene dyes, i.e., Erythrosin B (EB), Eosin Yellowish (EY), Phloxine B (PB) and Rose Bengal (RB).

The reflection or transmission properties of a material as a function of wavelength are measured quantitatively by spectrophotometry, which deals with the amount of chemical substance that absorbs the light by measuring the attenuation of light intensity as a beam passed through the sample solution. This means a device measures the amount of photons absorbed that passed through sample solution. This measurement is used in many quantitative applications in various fields, for example chemistry, physics, biology, biochemistry, material and chemical engineering, clinical applications, industrial applications, etc. [88].

**Figure 2:**
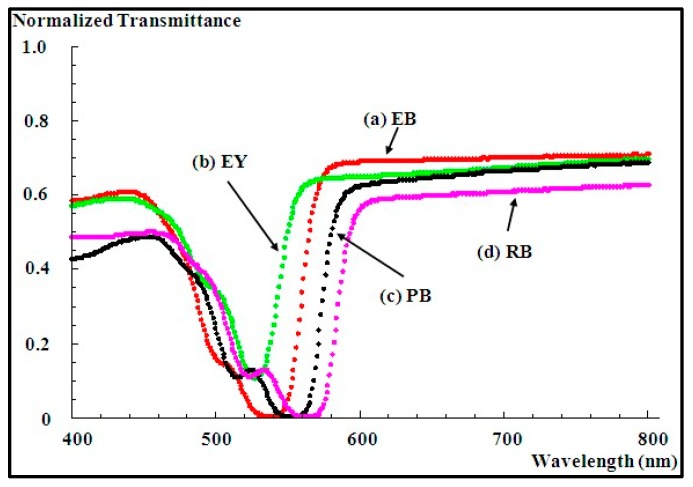
Normalize transmittance spectrum for four different photosensitizers (**a**) EB (red curve); (**b**) EY (green curve); (**c**) PB (black curve); and (**d**) RB (pink curve) in AA/PVA photopolymer material [89].

For example, in our photopolymerization process, we presented the absorbed spectrum over the range of visible wavelengths in Figure 2 for our four dyes. Efficient absorption by a dye determines the wavelength of best photosensitivity. The purpose of these examinations is giving us more understanding of the behavior of these dyes over a range of wavelengths during the process of polymerization. As can be seen, these dyes are much more active (react) over light wavelengths ranging 510–580 nm.

**Figure 3:**
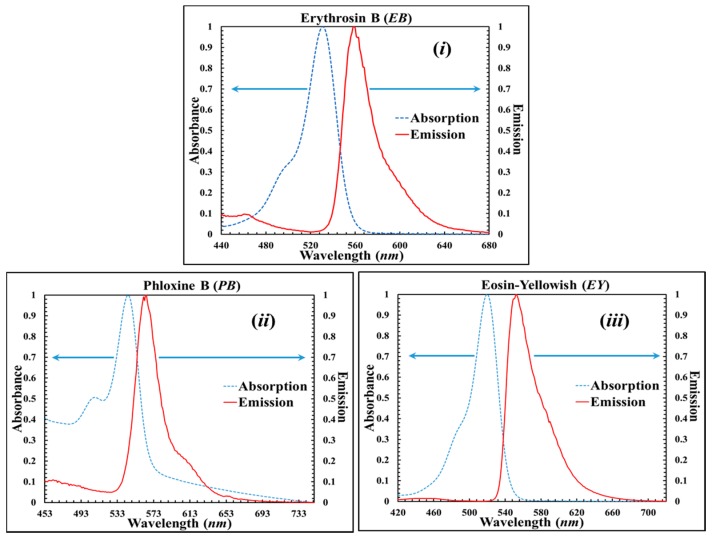
Normalized of absorbance (blue-dash curve) and emission (red curve) spectra plotted against wavelength for AA/PVA photopolymer material including different dyes: (**i**) EB; (**ii**) PB; and (**iii**) EY.

To gain a better understanding of the reactions of the dyes, we examined the UV–visible absorption spectra of AA/PVA solutions (including different Xanthene dyes, 1.25 × 10^−3^ M), which were acquired using a double beam (Jasco V-650, Easton, PA, USA) Spectrophotometer. Spectra were recorded over the range of 300–900 nm. Solution sample measurements were carried out using quartz 1 mL cuvette (Hellma, Jena, Germany). Photoluminescence (PL) spectra of the solution were acquired using a (Q-40; PTI (Photon Technology International, Birmingham, NJ, USA)). Felix GX 4.1.0 software was used for data acquisition. Emission spectra were recorded from absorbance λ_max_ + 10 nm for the samples up to 900 nm, with slit widths of 4.5 nm, and scans speed of 1 nm·s^−1^. Typically, light emission (by an atom or molecule) occurs some finite time subsequent to the absorption of the incident electromagnetic energy and is at a longer wavelength. Absorption involves an electronic transition that promotes an electron from the ground state to an unoccupied orbital [90]. An experimental measurement (excites) result, using the system described above, is presented in Figure 3, which illustrates the normalized excitation and corresponding emission spectra, for different dyes, overlaid on the same graph. The emission spectrum is almost a “mirror image” of the absorption spectrum. The solutions examined contained the standard photopolymer material, including different dyes, e.g., (i) B (EB); (ii) B (PB); and (iii) (EY). As can be seen in Figure 3, all of these dyes are sensitive to (i.e., absorb) light of wavelengths around 532 nm. In our experiments, we typically use a green laser, λ = 532 nm.

These results are important in our study, which discussed the general characteristics of the dyes used to photosensitize our layer. We now describe the material composition. The AA/PVA photopolymer material is made using the components listed in Table 1.

It is prepared using the following steps:

(a) Ten grams of PVA was added to 100 cm^3^ of deionized water and dissolved using a heater/stirrer. This solution is then allowed to cool and 70 cm^3^ of this solution is transferred into a beaker.
(b) Triethanolamine (TEA) (8 cm^3^) electron donor was added to the PVA solution and stirred thoroughly.
(c) Acrylamide (AA) (2.4 g) and bis-acrylamide (BA) (0.8 g) were added to the PVA solution under a fume cupboard conditions and stirred until completely dissolved.
(d) For green photosensitive material, 16 cm^3^ of 1.25 × 10^−^^3^ M Xanthene dye is added to the beaker. This step and subsequent steps were carried out under a red safety light, as the material is now sensitive to green light.
(e) The solution is then made up to 100 cm^3^ in a volumetric flask with deionized water.
(f) The solution is then stored in the dark ready for plate preparation.

To prepare dry material layers for a Holographic recording medium, the solution prepared above, is used as follows:

(1) The glass substrate on which the material is to be deposited (typically 5 cm by 4 cm or a standard microscope slide) is cleaned thoroughly using deionized water and Acetone. Then, the plates are placed on a level surface so that the photopolymer layers would adhere to the glass evenly to produce a layer of uniform thickness.
(2) The photopolymer solution, 2 mL, is deposited evenly over the area of the glass plate using a normal syringe.
(3) This method is used to prepare a typical material thickness (100 ± 10 μm). In addition, we can obtained different thicknesses by depositing (drop casting) different quantities of material. By using a micrometer screw gauge, we can measure the thickness the thickness and uniformity of these layers.
(4) The plates are then left in the dark for approximately 24 h until dry to be ready for use.

The Self-Written Waveguides (SWWs) studies are discussed in our work. In this case, it is necessary to prepare much larger and denser AA/PVA material (samples), and a different technique (different to that used to prepare layers), involving a carful process of heating and drying, must be employed. To prepare solid bulk material samples for our SWWs studies the solution prepared above is used as follows:

(1) To evaporate the water from AA/PVA solution, we heated it while keeping it continuously agitated using a conventional magnetic stirrer. This process was performed under safe light and stable laboratory environmental conditions, with keeping AA/PVA solution at a constant temperature less than 100 °C.
(2) After most of the water content evaporated, which is done for around 6 h per 100 mL, the hot solution (high viscosity liquid) is quickly poured into cuvettes (12.5 × 12.5 × 45 mm^3^).
(3) Then, the cuvettes were rapidly transferred to a low pressure chamber to eliminate the air bubbles present in the AA/PVA, and also to allow uniform cooling of the hot material under the low temperature conditions produced under low air pressure.
(4) These samples were stored in the dark for a long period (several days) to allow them to stabilize and to allow any remaining water to completely evaporate.
(5) Following that, a dry AA/PVA was extracted from the cuvettes using a tweezers. Finally, we produced samples of size (*x* × *y* × *z*) 8.5 mm × 10 mm × 8.5 mm, which is suitable for use in performing self-writing experiments.

## 3. Holographic Gratings

### 3.1. Introduction to Holography

In recent years, advances in the area of optics have resulted in an ever increasing demand for holographic solutions for a range of technical applications. In this section, we introduce a brief review of diffraction by a holographic grating. In conventional photography, one is concerned only with the irradiance distribution of an image. Information regarding the relative optical path to different parts of the object are not recorded, as the photographic emulsion is a square law detector and records only the amplitude of the irradiance. In the case of holography, a complex light field, both the amplitude and phase of an object, can be recorded and reconstructed. Holography is in essence a technique for recording the full optical field.

In 1948, Dennis Gabor first described the imaging technique known as holography [91]. Gabor outlined a method to capture/record a three dimensional image of an object by interfering a set of mutually coherent light waves at a photographic plate. Holography is a two-step process.

**Figure 4:**
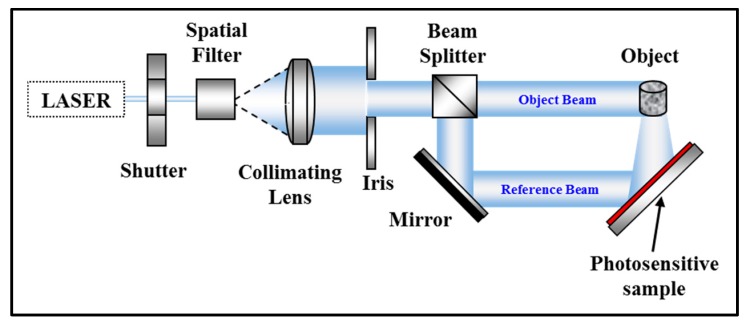
Basic set-up for recording an off axis transmission type hologram.

The first step: referred to as the exposure or recording step, involves the fabrication of the hologram (see Figure 4). In this case, an information carrying field, i.e., a wavefield reflected from an object, overlaps at a holographic plate with a second field known as the reference wave, resulting in an interference pattern, which is captured or recorded by the light sensitive medium.

**Figure 5:**
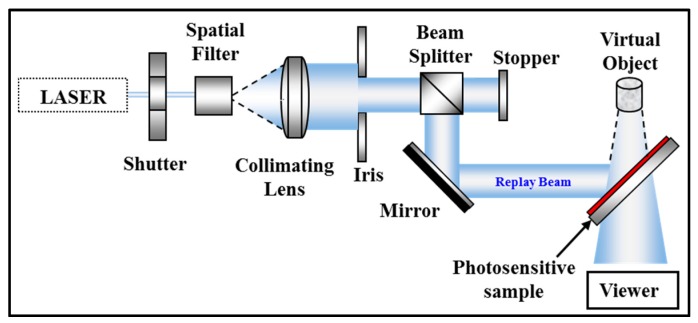
Basic set-up for reconstruction an off-axis transmission type hologram.

The original second step: referred to as the reconstruction or replay step, involves the recreation of the exposing wavefield. This is achieved by illuminating the recorded image using a beam, corresponding to the original reference wave, to reproduce the original object wavefield. The holographic construction step can be seen in Figure 5.

Gabor implemented this new process of wavefront recording using a mercury discharge lamp and collinear (in line) object and reference beams. However, the availability of coherent monochromatic light source and the use of an off-axis geometry are necessary in the creation of practical holograms to establish a fixed phase relation between the object and the reference wave at every point, and to separate the transmitted and diffracted light. It was not until the 1960s, with the development of the laser, that holography began to make significant technical advances [92].

### 3.2. Diffraction by Thin Gratings

**Figure 6:**
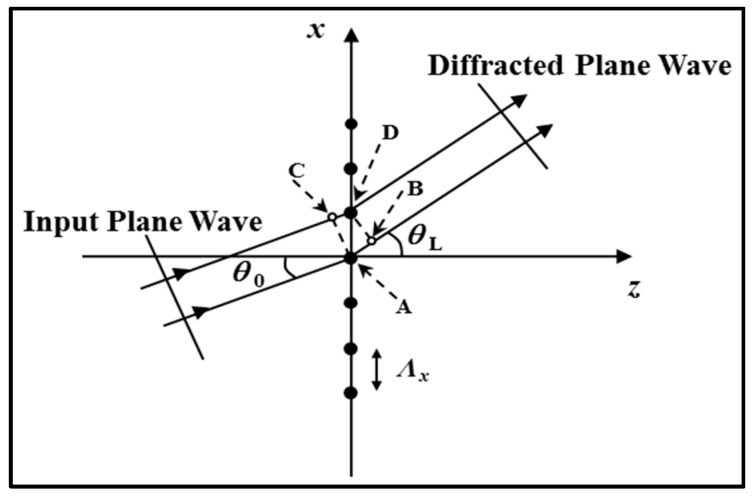
Schematic of diffraction by a thin transmission grating, following [19].

We begin by discussing simple thin transmission gratings. Figure 6 shows a two-dimensional periodic structure made up of an infinite array of point scatterers, spaced Λ*_x_* apart [19]. Our aim is to examine the effect of the grating on an incident (input) plane wave. It is assumed that the incident wave is infinite, monochromatic and has free space wavelength λ. The wave is incident at an angle $\theta_{0}$ to the z-axis and the refractive index *n* is uniform throughout the medium, so the effective wavelength of the light in the medium is λ/n.

Due to the grating periodicity, plane waves emerge from the grating at discrete angles. This is a result of constructive and destructive interference occurring as light is scattered by the periodic grating. Constructive interference will occur when the difference between the path lengths *AB* and *CD* is a whole number of wavelengths. This will happen at an output angle$\;\theta_{L}$, if

(1) $$AB-CD=\Lambda_{x}\left(\sin\theta_{L}-\sin\theta_{0}\right)$$

where *AB* *−* *CD = L*λ/*n* and *L* is an integer therefore

(2) $$\sin\theta_{L}=\sin\theta_{0}+L\lambda /\left(n\Lambda_{x}\right)$$

Equation (2) is known as the (Fraunhofer) grating equation. For an incident plane wave at a given incident angle,${\;\theta }_{0},$ a number of output plane waves are generated for discrete diffraction orders. For the *L*th diffracted order, the angle of diffraction,${\;\theta }_{L}$, can be determined by solving Equation (2). It is clear that the angle of diffraction is dependent on the wavelength of the incident plane wave, λ, and the period of the grating, Λ*_x_*.

To achieve a larger angular separation of the diffracted orders, smaller grating periods or a larger replay wavelength must be used. Note that the grating equation reveals nothing about the intensities of the diffracted orders. This type of thin transmission grating is unselective, favoring no particular incidence condition or diffraction order [19,93].

### 3.3. Thick Volume Gratings

**Figure 7:**
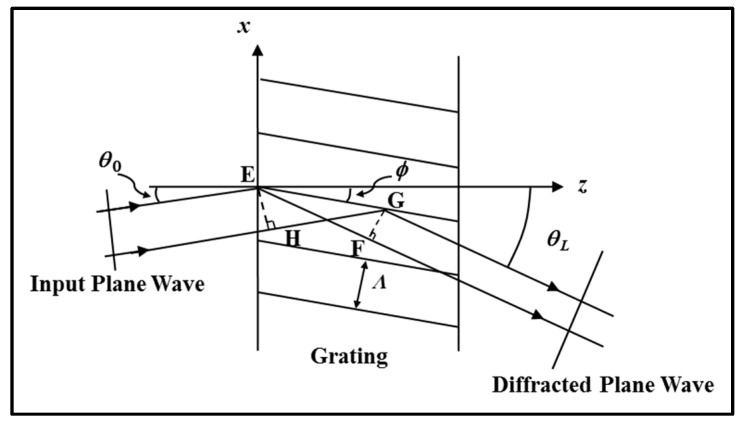
Schematic of a volume transmission grating, following [19].

Thin transmission gratings produce a large number of forward propagating diffraction orders. To reduce the number of diffracted orders containing significant light intensity, and to introduce greater angular and wavelength selectivity, grating fringes, i.e., extended scatters, must exist, as shown in Figure 7 [19]. In this case, the fringes are slanted and the grating is periodic in *x* and *z*. φ is the slant angle and Λ*_x_* = Λ/cosφ. Scattering is now extended in the *z*-direction. The grating equation, Equation (2), is still valid but now an extra constraint is placed on the diffraction process. This scattering process is illustrated in Figure 7.

If it is assumed that constructive interference only occurs if all the scattered contributions add in phase, then the path lengths *EF* and *HG* must be equal. This means that some diffraction angles are preferred. These angles are defined by:

(3) $$\theta_{L}=\theta_{0},{\;\mathrm{or}\;\theta }_{L}=2\phi -\theta_{0}$$

If $\;\theta_{L}=\;\theta_{0}\;$then the only solution to Equation (2) is *L* = 0, the zero^th^ transmitted diffraction order. If $\theta_{L}=2\phi -\;\theta_{0}\;$then,

(4) $$\sin\left(2\phi -\theta_{0}\right)-\sin\theta_{0}=2\sin\left(\phi -\theta_{0}\right)\cos\phi =\frac{L\lambda }{n\Lambda_{x}}=\frac{L\lambda \cos\phi }{n\Lambda }$$

giving the Bragg condition as

(5) $$2\Lambda \sin\left(\phi -\theta_{0}\right)=L\lambda /n$$

Therefore, for example, the –1st order occurs when

(6) $$2\Lambda \sin\left(\phi -\theta_{0}\right)=\lambda /n$$

which is the well-known Braggs Law of diffraction [19]. Higher order Bragg angles can also be determined. For an incident beam at the *L*th Bragg angle the transmitted (zeroth order) beam and the *–L*th diffracted beam satisfy the Bragg condition. Whether a grating behaves more like a thin regime grating or more like a thick (volume) regime grating, depends on parameters such as the layer thickness, index modulation and grating period. Differentiation is possible using *Q* and Ω parameters [19] defined by

(7) $$Q={\left|K\right|}^{2}d/\beta ,\;\mathrm{and}\;\Omega ={\left|K\right|}^{2}n/\beta n_{1}$$

Their values can be used to identify the type of grating being analyzed. In Equation (7), *d* is the grating thickness, *n*_1_ is the amplitude of the grating refractive index modulation, |***K***| = 2π/Λ is the grating vector magnitude and β = 2π*n*/λ is the propagation phase constant. If *Q* >> 1 and Ω > 20, the grating is considered to be a thick volume grating capable of exhibiting high diffraction efficiency (strong Bragg scattering). The Bragg condition itself tells us nothing about the diffracted intensity values (beyond that it can be large).

Using the grating and Bragg equations, we have shown how the input wavelength, grating thickness and grating period determine the replay angle at which incident light is diffracted into the 1st diffracted order. In this article, we will be primarily dealing specifically with unslanted (φ = 0) volume transmission type holographic gratings recorded and replayed using plane waves.

### 3.4. Volume Holographic Gratings

The simplest form of a hologram is Volume Holographic Grating (VHG). A VHG is defined as a material, in which the permittivity, ε, and/or conductivity, σ, vares continuously in space in a nearly periodic manner [93]. If only the permittivity varies, it is known as a phase grating and, if only the conductivity changes, it is known as an absorption or amplitude grating. If both vary, the grating is known as mixed.

**Figure 8:**
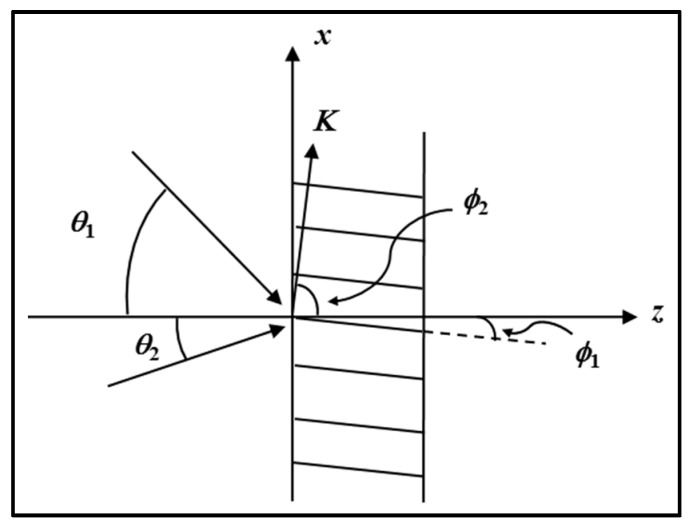
Volume Holographic Grating (VHG) recording set-up, where ***K*** is the grating vector (perpendicular to the fringes) and $\phi_{1}\;$is the grating slant angle.

Let a simple sinusoidal VHG be recorded using plane waves for both the object and reference fields. If the two waves are incident at angles $\theta_{1}\;$and$\;\theta_{2}$ (as shown in Figure 8), their complex amplitudes can be written in the form:

(8) $$E_{i}=A_{i}\exp\left(-j\rho_{i}\cdot r\right),\;\mathrm{where}\;i\;=\;1,\;2,\;\cdots$$

where the propagation vectors $\rho_{1}\;$and $\rho_{2}\;$are defined as $\rho_{1}=\beta \left(-\sin\theta_{1}a_{x}+\cos\theta_{1}a_{z}\right)\;$and $\rho_{2}=\beta \left(-\sin\theta_{2}a_{x}+\cos\theta_{2}a_{z}\right)$, with **a***_x_* and **a***_z_* the unit vectors in the *x* and *z* directions, respectively, and ***r*** = (*x*, *z*) is the displacement vector. The recording geometry is illustrated in Figure 8.

As mentioned earlier we first assume the photosensitive material responds to the intensity of the interference pattern. The intensity is proportional to the magnitude of the sum of *E*_1_ and *E*_2_ electric field amplitudes. The intensity distribution at the plate is therefore given by

(9) $$\begin{array}{ll} I_{\exp}=E_{Total}E_{Total}{}^{*} & =\left(E_{1}+E_{2}\right){\left(E_{1}+E_{2}\right)}^{*}\\ & =A_{1}^{2}+A_{2}^{2}+2A_{1}A_{2}\cos\left[\left(\rho_{1}-\rho_{2}\right)\cdot r\right] \end{array}$$

where

(10) $$\rho_{1}-\rho_{2}=-2\beta \sin\phi_{2}\left(\cos\phi_{1}a_{x}+\sin\phi_{1}a_{z}\right)$$

with$\;\phi_{1}=\left(\theta_{1}-\theta_{2}\right)/2,$ and $\phi_{2}=\left(\theta_{1}+\theta_{2}\right)/2$. Equation (9) contains three terms, the first two being the sum of the squares of the field amplitudes and the third a periodic cross term whose amplitude is proportional to their product (cosinusoidal intensity distribution at the plate). If this term is recorded, the result will be a pattern of regular cosinusoidal fringes extending through the photosensitive material volume. If a phase only grating is formed, and it is assumed that the permittivity change in the materials is linearly proportional to the exposure energy (i.e., the product of the irradiance, *I*_exp_, and the exposure duration or time, *t*), the resulting change in permittivity can be written as

(11) $$\Delta \varepsilon_{r}=k\times t\times I_{\exp}=kt\left\{A_{1}^{2}+A_{2}^{2}+2A_{1}A_{2}\cos\left[\left(\rho_{1}-\rho_{2}\right)\cdot r\right]\right\}$$

where *k* is a material response constant. Examining Equation (11), it can be seen that the resulting change in the relative permittivity distribution will have the form:

(12) $$\Delta \varepsilon_{r}=\varepsilon_{r0}+\varepsilon_{r1}\cos\left(K\cdot r\right)$$

where ***K*** is the grating vector, whose direction lies perpendicular to the lines of constant permittivity (grating fringes), with ***K*** = 2π/**Λ**, where **Λ** is the period of the grating. The parameter ε*_r_*_0_ is an average constant change of permittivity, and ε*_r_*_1_ is the amplitude of the permittivity modulation. Comparing Equations (11) and (12), we note that:

(13) $$\varepsilon_{r0}=kt\left(A_{1}^{2}+A_{2}^{2}\right),{\;\mathrm{and}\;\varepsilon }_{r1}=2ktA_{1}A_{2}$$

and that

(14) $$K=\rho_{1}-\rho_{2}=-2\beta \sin\phi_{2}\left(\cos\phi_{1}a_{x}+\sin\phi_{1}a_{z}\right)$$

In Figure 8, we see that the ***K*** vector is oriented perpendicular to the fringe planes, $\phi_{2}\;$is the angle between the ***K*** vector and the normal to the layer plate surface and $\phi_{1}\;$is the grating slant angle. From Equation (14), we can determine the fringe spacing.

(15) $$\Lambda =\frac{2\pi }{\left|K\right|}=\frac{\pi }{\beta \sin\phi_{2}}=\frac{\lambda }{2n\sin\phi_{2}}$$

For symmetric exposure $\theta_{1}=\theta_{2}$, if an unslanted transmission grating is recorded, in which $\phi_{2}=\pi /2$ and $\phi_{1}=0,$ giving ***K*** = 2βsin$\theta_{1}$**a***_x_*, Equation (15) reduces to:

(16) $$\Lambda =\frac{\lambda }{2n\sin\theta_{1}}$$

**Figure 9:**
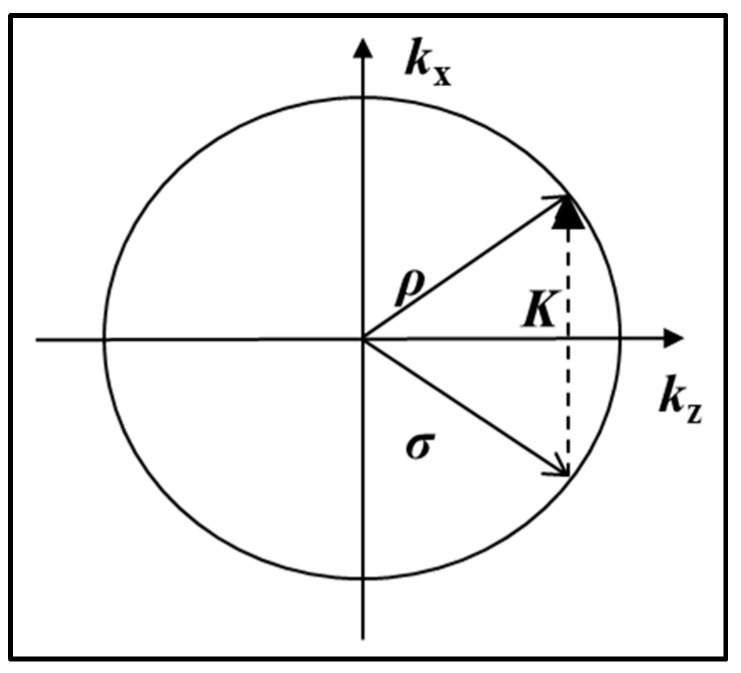
Ewald diagram of an unslanted transmission grating replayed the on-Bragg angle, **σ *= ρ*** − ***K***. *k*_x_ and *k*_z_ are the *x* and *z* phase space axes.

Figure 9 shows the Ewald diagram of such an unslanted transmission grating being replayed on-Bragg, i.e., at the Bragg angle (satisfying the Bragg condition), **σ *= ρ*** − ***K***. In Figure 9, ***ρ*** and **σ** are the propagation vectors of the transmitted and first order diffracted waves inside the grating and ***K*** is the grating vector.

**Figure 10:**
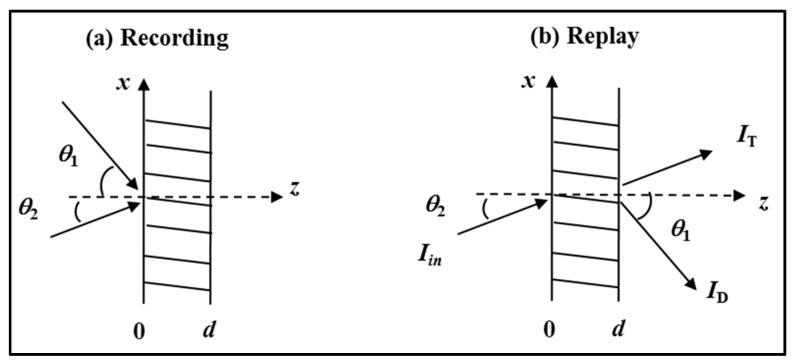
(**a**) Recording (exposing); and (**b**) replay of a volume transmission gratings. *I*_in_ is the incident intensity of the replay wave, *and I*_T_ and *I*_D_ denote the transmitted and diffracted intensities, respectively.

Once the grating has been recorded, some material post-processing may be necessary before replay. In the case of the photopolymer systems studied in this article, no such post-processing is necessary. Replaying the grating using the original reference wave, incident at an angle$\;\theta_{2},$ will reproduce the object wave by means of diffraction, as shown in Figure 10b.

This type of grating geometry is used throughout work to characterize photopolymer materials and is known as a transmission grating. It should be noted that, in an unslanted grating, the fringes are perpendicular to the surface of the material, and so any process that results in changes in the thickness of the material layer does not affect the period of the fringes. Experimental angular scans of unslanted transmission gratings (which involve measuring changes in the diffraction efficiency as a function of the angle of replay) verify that no change in the value of the on-Bragg angle (replay condition) occurs as the material is exposed [94].

In summary, in this subsection, we have briefly described how the interference of two plane waves generates a sinusoidal intensity pattern. Illuminating a suitable photosensitive material in this way, results in a sinusoidal modulation of the material’s permittivity, leading to the recording of a holographic phase (lossless) grating. In the photopolymer materials discussed in this article, the modulation in the material’s permittivity occurs during holographic exposure, i.e., the material is non-latent [19] and requires no post processing, i.e., it is self-processing. Much of the work of this article involves more fully understanding the grating formation processes that occur, during both recording and post-exposure.

### 3.5. Refractive Index Modulation

In the study of self-processing holographic recording materials it is common to simultaneously record gratings in photosensitive materials, i.e., photopolymer materials, and to also simultaneously optically examine, or probe, the resulting gratings. In this way real time measurement of the material evolution during exposure can be performed. The modeling of diffraction by the gratings during replay presented in this article is based on Kogelnik’s two-wave coupled wave theory [20].

This model presented here describes the diffraction efficiency of thick volume holograms VHGs. It was shown that during replaying the hologram, incident beam deviates from the Bragg condition. Thus, the analytic expressions for both the angular and wavelength dependence of the diffraction efficiency can be derived. In addition, time dependence of the diffraction efficiency, ŋ(*t*), as a function of a number of grating parameters can be described. For a lossless, unslanted transmission geometry grating, replayed on-Bragg with TE polarized probe light, for example see [20,95], ŋ(*t*) is described as,

(17) $$\eta \left(t\right)=\frac{I_{D}\left(t\right)}{I_{in}}=\sin^{2}\left[\frac{\pi n_{1}\left(t\right)d}{\lambda \cos\theta_{B}}\right]$$

where *I*_in_ and *I*_D_(*t*) are the incident replay and time varying diffracted probe beam intensities, respectively. As before, *d* represents the grating thickness, while θ_*B*_ and λ are the Bragg angle and the wavelength of the incident probe beam inside the grating, respectively. Importantly, *n*_1_(*t*) is the grating refractive index modulation, i.e., the refractive index amplitude of the grating. In deriving Equation (17), all boundary reflections are neglected. Therefore, we can approximate the diffraction efficiency of an unslanted transmission holographic grating using the diffraction selectivity:

(18) $$\eta \left(t\right)=\;\frac{I_{D}\left(t\right)}{I_{D}\left(t\right)+I_{T}\left(t\right)}$$

where *I*_D_ is defined to be the measured temporal evolution of the resulting Fresnel corrected first-order diffracted intensity and *I*_T_ is the corresponding corrected transmitted intensity of the probe beam. Rearranging Equation (17), an expression for the temporally varying refractive index modulation, *n*_1_(*t*), can be obtained from the experimentally measured intensity values,

(19) $$n_{1}\left(t\right)=\frac{\lambda \cos\theta_{B}}{\pi d}\sin^{-1}\left[\sqrt{\eta \left(t\right)}\right]$$

The grating is typically replayed on-Bragg during exposure using a low intensity probe beam having a different wavelength that to which the photopolymer is exposed. Meaning the wavelength of probe beam is a different from the wavelength of recording beams. Using Equation (19), and the measured transmitted and diffracted intensities of the probe beam, growth curves showing the refractive index modulation growth as a function of exposure time, i.e., *n*_1_(*t*), can be extracted.

We should note that a rigorous coupled-wave approach [64] can be used to analyze diffraction by general planar gratings bounded by two different dielectric media, e.g., air and a glass substrate. It should also be noted that the results for off-Bragg replay can be predicted with higher accuracy using the Beta-value expression of Uchida [96,97,98] rather than Kogelnik’s model.

### 3.6. The Grating Formation Process

**Figure 11:**
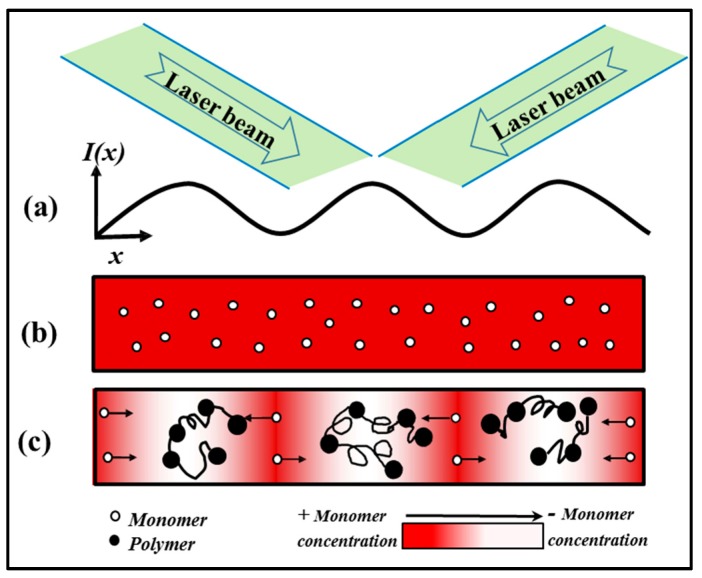
Formation of grating in a photopolymer material: (**a**) the sinusoidal illuminating intensity distribution at the plate; (**b**) the uniform photopolymer before recording; and (**c**) the photopolymer during recording (mass transport and polymer chains).

The grating formation process in a photopolymer material is illustrated in Figure 11. The sinusoidal illumination intensity pattern causes polymerization which takes place most strongly in the regions of high intensity exposure. Most dye radicals are generated in areas most strongly illuminated, leading to the greatest amount of initiation of chain growth. In these regions, the monomer is consumed due to polymerization, and also a monomer concentration gradient is created. Figure 11c shows the excess monomer in the weakly illuminated regions begins to diffuse and mass transport into the brighter regions to eliminate the concentration gradient.

This results in the formation of a permanent sinusoidal polymer concentration spatial distribution. Assuming all the monomer is converted to polymer by the end of the recording process, local variations of the permittivity of the material will be present which will be proportional to the fixed local polymer concentration. Thus, a modulation of the permittivity exists, creating a grating of the form described by Equation (12).

## 4. Self-Written Waveguides (SWWs)

It has been known and demonstrated for many years that SWWs can be fabricated in nonlinear and self-processing materials, including in photopolymers [99,100]. The second part of the work performed in this paper involves an experimental and theoretical study of the self-writing process in photopolymer material, both in bulk materials and in planar layers. We start here by giving a brief review of Self-Written Waveguides in Section 4.1. Some previous studies and the related applications are discussed in Section 4.2.

### 4.1. Introduction to the Self-Writing Process

Self-writing is a technique of directly fabricating waveguide structures within a material. In recent years, it has been demonstrated that SWWs can form in a variety of nonlinear materials, i.e., Kerr medium [101], photosensitive glass [102] and photopolymer material [99,100]. In all of these cases, the exposing light induces some refractive index change, which take place sufficiently rapidly to then act on the exposing beam. In this subsection, an overview this process is given.

**Figure 12:**
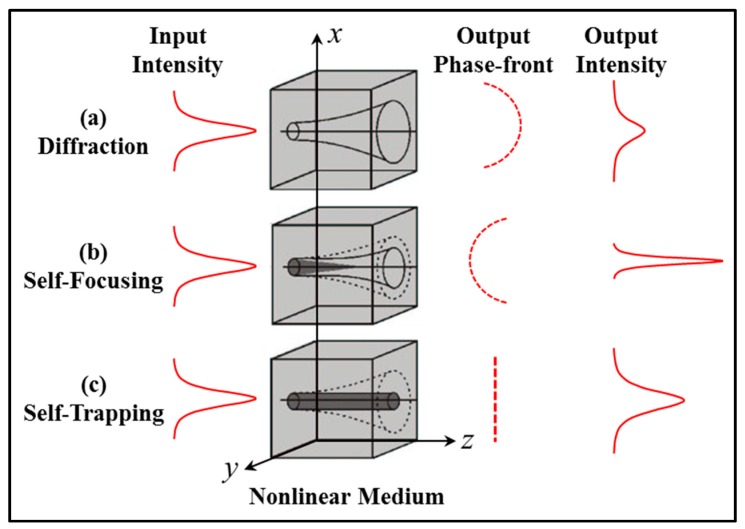
Schematic illustrations of the spatial beam profiles, output phase-front, and output intensity of a propagating beam as it undergoes: (**a**) natural diffraction; (**b**) self-focusing; and (**c**) self-trapping.

Consider a focused Gaussian beam, whose waist is incident on the input boundary of a photosensitive sample having an initial uniform refractive index. Initially, the light diffracts freely though the homogenous material as any induced index changes will not take place instantaneously (see Figure 12a). Meanwhile, this incident light intensity distribution will start to change the refractive index within the material. Once self-focusing begins, the induced changes in the refractive index (typically involving an increase in index where the intensity is highest) start to compensate for the light diffraction spreading (as the light propagates). As the refractive index increases along the propagation axis, a channel begins to be formed and a self-focused taper is created within the material (see Figure 12b). The induced channel evolves into a waveguide and the diffraction effects may be exactly balanced by the optically generated self-focusing effects. Thus, eventually the self-written waveguide acts to eliminate beam diffraction in the material. The associated nonlinear optical phenomena have been widely studied and the effect is referred as self-trapping [103,104,105] (see Figure 12c).

As indicated during waveguide evolution, the diffraction spreading of the writing beam is overcome by the induced refractive index change in the medium. It should be noted that in most cases previously studied, significant diffraction through the material is necessary in order that the light intensity (at the input edge), can induce enough refractive index change to form the waveguide. This is the case for many photosensitive materials including photosensitive glasses [8,102]. Therefore, photopolymer materials have many significant advantages when used to fabricate self-written waveguide channels. For example, the response time for photopolymers is rapid and the resulting refractive index change is relatively large. It is known that exposure times of the order of minutes or seconds are necessary to produce a saturation refractive index change as large as ~0.04 in a liquid photopolymer [99,100]. Typically, photo-polymerization processes are a function of the absorbed energy rather than being produced by a nonlinear intensity response. Therefore, photopolymer materials have a high sensitivity requiring relatively low exposure times [12,13,106]. The index changes produced in photopolymers due to the photo-polymerization process have good lifetimes (i.e., are fixed stable) under normal conditions [11,37]. Therefore, the resulting self-written channels can be treated as being permanent. Furthermore, the sensitivity, spectral photo-response and refractive index change the capability of a given photopolymer system relatively easily by adjusting the concentrations and types of dye used during photopolymer preparation, is also controlled [17,89,107,108,109,110].

As noted, in Figure 12, a Gaussian writing beam is used to illustrate the production of a channel waveguide. If a different writing pattern is applied, i.e., a different beam shape, energy or wavelength, more complicated refractive index structures can be self-written. For example, when two incident beams collide with each other (e.g., cross) within a photo-polymerizable resin, they can merge to produce a single waveguide and single output beam [111]. When exposing light is introduced via a single-mode optical fiber and a very low beam power is used, a unique uniform-channel waveguide can be obtained by photo-polymerization. However, if the input power is increased, the guide pattern will become chaotic and multichannel [106]. If incoherent white light is used, the independent formation of the filaments was generated through self-trapping of incandescent speckles, the dependent filaments created through modulation instability of a broad incandescent beam have been also observed [112].

This discussion of the formation of self-written waveguides illustrates that the self-writing process is a versatile technique in which permanent waveguides can be created directly in the material. We note that the optical self-writing techniques described above are significantly different to more traditional waveguide fabrication methods, employed for example on etching, additive lithography or photoresist based techniques [113,114].

### 4.2. Previous Work on Self-Written Waveguides

In recent years, different types of photopolymer materials have been used to explore the light self-writing technique, irradiating using coherent laser light [13,115,116,117,118], and partially coherent/incoherent light sources [112,119,120,121]. The materials examined have been prepared as either liquid solutions [11,99,100,106,122] or solid samples [14,15,123,124] for a range of possible applications. In this subsection, a brief overview of the research reported to date in this area is presented.

Kewitsch and Yariv [99] were the first to theoretically and experimentally demonstrate that permanent self-written optical channel structures can form using photo-polymerization. Initially in this case the chains formed are relatively short and the increase in material density was observed to be insufficient to cause any significant index change. As the polymerization progresses, longer chains are formed, increasing the sizes of the refractive index changes. It was reported [99] that, for two liquid photopolymers, i.e., a diacrylate and triacrylate, the maximum index changes are 0.043 and 0.028, respectively. For the diacrylate photopolymer, the beam was guided over a distance greater than 10 mm by illuminating the polymer at wavelength 325 nm. The resulting nonlinear wave equation is also presented. It is shown to be nonlocal in time and displays self-trapped solutions only for sufficiently low average exposing optical intensities. The results also indicated the wave slow time response associated with waveguide formation.

In the same year, Ref. [100] also proposed using the self-focusing and self-trapping phenomenon in projection photolithography, in order to enhance the recording resolution and depth of focus. In the theory presented the propagation of the beam through the exposed photopolymer is modeled using the beam propagation method and self-focusing is demonstrated.

In 1998, Monro et al. [125] carried out the analysis of self-written waveguide formation in both planar and bulk waveguide geometries, with both experimental and theoretical results being represented. A series expansion technique was used to describe the refractive index change induced during the self-writing process. The result was described using a simple approximate phenomenological model. It is relatively straightforward to apply this approach to other self-writing processes, i.e., photosensitive glasses or photopolymers, so long as the index evolution can be well described using the proposed phenomenological expression. This is necessary to derive the necessary recurrence relations. When the parameter values used in the model expression are carefully chosen, the numerical predictions have been shown to agree well with the experimental results.

To deal in a quantitative manner with the chemical parameters of the material and to study the diffusion of the chemical reactants present, Yariv’s group, i.e., Engin, Kewitsch and Yariv [126], also developed a holographic characterization technique. In this way, the photo-polymerization behavior of three difunctional acrylate monomers were examined, i.e., TMPTA (from UCB Radcure), butyle ethylene and HDODA (UCB Radcure). In all cases a 0.1 wt % initiator concentration (Irgacure 369) was used. Using this technique, they were able to determine the lowest value for the largest value of the diffusion constant in the multifunctional acrylates. Significantly, we note that, while the study in this analysis involved holographic characterization of chain photo-polymerization, the results were used to establish the basis of a theory in order to allow the further investigation of self-written waveguides. This work is instructive, as it showed that improvements in the material model is possible, but require a more accurate description of the photochemical reactions taking place during the self-writing process.

Shoji and Kawata [127] examined the interactions of optically-induced growth of fiber like patterns into a photo-polymerizable resin. Optical growth of a single or multiple fibers was achieved by focusing ultraviolet (UV) laser light into the photo-polymerizable resin. A liquid photopolymer solution was exposed, which consisted of urethane acrylate monomers and urethane acrylate oligomers, as well as photo-initiators for the photo-polymerization reaction. A He–Cd laser operating at 441.6 nm was used to illuminate the material solution. In the experiments, they observed that two optically grown independent fibers could merge to form a single fiber under specific conditions. One example of a particular set of such conditions occurred, when the two beams are launched simultaneously with a power of 0.1 mW, an input beam width of 0.96 μm, and the angle between the converging beams was adjusted to be less than 9°. In that case, it was shown that the self-written waveguides merged into one channel. However, if a larger angle was used, no merging occurred and the two waveguides evolved separately. If the power ratio between the two lasers was altered, the direction of the waveguide after merging was tilted toward the optical channel of the beam with the larger power.

Eldada and Lawrence [128] reported that planar single-mode, multimode, and micrometer-sized waveguide structures (ranging in dimensions) were produced in photopolymers, especially in solution or when using liquid monomers. Transmission losses in such polymer waveguides could be minimized, typically by halogenation, with state-of-the-art loss values being about 0.01 dB/cm at 840 nm and about 0.1 dB/cm at 1550 nm. These material properties enabled fabrication of a variety of optical waveguide devices, which include straight waveguides, bends, splitters, directional couplers, MMI couplers, star couplers, wavelength filters, and long, high-density waveguide arrays. The resulting devices could be fabricated to match the dimensions and numerical apertures of conventional optical fibers, meeting all practical application requirements.

In 2001, Kagami et al. [115] reported that 3D optical waveguides can be fabricated in a photo-polymerizing resin mixture solution using a multimode fiber. During exposure, a straight waveguide, with a length of 20 mm or more, was observed to grow by the self-trapping of a guided laser beam, using an exposing wavelength of 488 nm. This waveguide was formed within a liquid solution consisting of a mixture of two kinds of photopolymers, denoted as Resin A and Resin B. Resin A was a radical type resin produced by blending an acrylic acid and a urethane–acrylate oligomer, which was used for the higher refractive index core. Resin B was a cationic-type fluorine inclusion epoxy which was used as a lower refractive index material. Their studies suggest that such Light-Induced Self-Written (LISW) technology could be of potential utility when automating the optical fiber connection and packaging processes by virtue of the simultaneous waveguide growth and the cladding-curing processes.

Later in 2001, an experimental method of manufacturing micrometer-sized polymer micro-tip structures at the extremity of both single mode and multimode optical fibers was reported by Bachelot et al. [122]. This was done by first depositing a drop of a liquid photo-polymerizable formulation on a cleaved fiber. This was then exposed using light of wavelength 514 nm, with an output intensity of 10 μW and an exposure time of 30 s. This light emerged from the fiber and induced the polymerization process. The photo-polymerizable formulation applied in experiments was made up of three basic components: a sensitizer dye, an amine cosynergist, and a multifunctional acrylate monomer, pentaerythritol triacrylate (used as received from the supplier). This forms the backbone of the polymer network. A numerical calculation method, similar to the one used by Monro et al*.* [125], consisting of an iterative Beam-Propagation Method (BPM), was developed into which photochemical effects were included. The model predicted how the influence of oxygen (inhibition) on the polymerization process could explain the shape of the tip extremity formed.

In 2002, numerical simulations, corresponding to the experimental Y-junction structure waveguides formations previously reported in [127], were performed by Shoji et al. [111]. The phenomenological model proposed by Monro et al. [125] was further developed and applied to describe the growth and interaction of multi-channel self-written waveguides in photo-polymerizable resins. In this analysis, it was assumed that there existed a light intensity threshold for the photopolymer material and that photo-polymerization could only occur at the specific positions where the light intensity exceeds a certain threshold. The predictions of this model were in good qualitative agreement with experimental results.

A considerable amount of work on such junctions has been carried out by Hirose et al. [129,130]. They used an acrylate type UV-curable resin, under UV illumination, to produce waveguides. The time varying coupling loss was monitored during the waveguides formation. The coupling loss variation was discussed for various combinations of gap and offset between the input and output fibers and the resulting refractive index distribution of the cross-sectioned waveguide was measured. Their experimental results clearly suggested that a self-written waveguide can be used to easily connect two optical fibers. For separations less than 1000 μm, the coupling loss significantly decreased after SWW formation even when some lateral offset exists. With 30 μm lateral misalignment, the coupling loss was only 1 dB, which 40 μm lateral misalignments still only leads to the rather low loss value of 2 dB.

Dorkenoo et al. [106] investigated the self-written channels and multichannel propagation inside a unique bulk photo-polymerizable material. The photopolymer material was a mixture of three main compounds: a sensitizer dye (Eosin Y: 2′,4′,5′,7′-tetrabromofluorescein disodium salt, 0.1 wt %), a cosensitizer (methyldiethanolamine at 5 wt %), and a multifunctional acrylate monomer (pentaerythritol triacrylate) that was the solvent of the two other products. Light at 514 nm from an argon laser was introduced into the medium by a 3-mm-core single mode optical fiber. At a very low beam power, i.e., ranging from 0 to 5 μW, a uniform-channel waveguide without any broadening was observed. However, when the input power was increased to 100 μW, the guide became chaotic and multichannel. It was observed that the output of the fiber acted as scattering center of the light (almost like a perfect homogenous point source), and the direction of the input light, i.e., the fiber orientation, had no influence on the structure of the filaments. At the output of the optical fiber, the light intensity was too high to be contained within one channel. The propagation of the light in the photopolymer materials was described using a paraxial wave equation approximation as proposed by Monro et al. [125]. In the paper, it is stated that the permanent properties of the waveguide induced in such photopolymers opened new possibilities for developing organic optical devices.

In 2004, Bachelot et al. [131] reported further work regarding the fabrication of polymer tips, integrated at the end of a 9-mm-core telecommunication optical fiber. The material preparation and the experimental setup used to fabricate the polymer tips are described in detail in their previous work [122]. In this work, it was shown that a high-efficiency coupling, 70% and 1.5 dB loss, between the fiber and an infrared laser diode, could be obtained. This result obtained by controlling the radius of curvature of the tip, the origin of which is discussed in terms of the photochemical effect of oxygen during tip formation. Carrying out simulations using a 2-D finite-element method (FEM), the harmonic solution is computed directly by solving the electromagnetic Helmholtz equation. The experimental results were found to be in good agreement with the rigorous electromagnetic calculations.

In 2005, a novel application of self-written waveguides in photopolymer materials was reported by Yonemura et al. [132]. In their study, a self-written waveguide module that provided bidirectional communication over a single plastic optical fiber with dual visible wavelength LEDs (green and red), was produced. The resulting low-cost polymer waveguide module was fabricated using light-induced self-written large diameter waveguides, which enabled a 3-D optical circuit for visible wavelength division multiplexing to be fabricated using an extremely simple process. The core of the waveguide was self-induced in a liquid photo-polymerizing resin (epoxy acrylate), which allowed a high refractive index of ~1.51. The exposing light used was produced by a diode-pumped solid state laser at a wavelength of 457 nm and a power of 5 mW. Using these modules and resulting measurements, the maximum transmission length, for both green (495 nm) and red (650 nm) LEDs, was estimated to be more than 20 m at a data rate of 250 Mbits/s in full duplex mode.

Hocine et al. [10] developed a new phenomenological model of the underlying photo-polymerization process to describe the growth of a polymer micro-tip on an optical fiber end. Compared to the theoretical model previously proposed by Monro et al. [125], this model was a significant improvement, and could more accurately simulate the polymer-component growth in a three-dimensional time-resolved manner. Therefore, the resulting numerical predictions more accurately describe and can thus be used to understand and optimize, the component growth conditions. This work focused on the role of oxygen either present (inhibition) in the atmosphere or dissolved in the solution. Their model is therefore more complete and physically realistic. First, a photo-polymerization rate empirical model was used to calculate the threshold energy, which related to the oxygen dissolved in the photopolymer solution and the local polymerization, during the inhibition and polymerization processes. Eventually, by coupling this with a standard Beam Propagation Method (BPM), a complete 3-D model of light propagation was developed. Furthermore, the decrease in absorption due to the consumption of the absorbing dye was considered and taken into account in the BPM process. In this way, the phenomenon of dye photo-bleaching was included in the algorithm. A set of quantitative experimental results was presented and used to find the values of the model parameters for the photosensitive solution. The solution used was made of three basic components: a sensitizer dye, an amine cosynergist, and a multifunctional acrylate monomer, pentaerythritoltriacrylate (PETIA) (used as received from the supplier). The cosynergist was methyldiethanolamine (MDEA), and Eosin Y (2′,4′,5′,7′-tetrabromofluorescein disodium salt) was used as the dye. Then the liquid photopolymer system was irradiated by light from: an Argon-ion laser (514 or 488 nm), a frequency-doubled Nd/YAG laser (532 nm), or a green He–Ne laser at 543.5 nm. The end-of-fiber optical components manufactured performed optical operations ranging from that of a simple lens to more complex functions.

In the same year, based on work to grow photopolymer micro-tips directly on the end faces of single-mode fibers, a novel method of light coupling between Single-Mode Fibers (SMFs) and small-core Photonic Crystal Fibers (PCFs) was demonstrated by Xiao et al. [133]. The single-mode fibers used were SMF-28 from Corning, and the small-core photonic crystal fibers used were NL-3.3-880 and LMA-5 PCFs from Crystal-Fiber A/S. The experimental method of manufacturing such self-growing photopolymer waveguides of micrometer-size, and the detailed principle of the photo-polymerizable formulation, have been detailed in the studies of Bachelot et al. [122,131]. Experiments showed that the coupling efficiency could be improved by up to 5 dB, compared with the results when no such micro-tips are used. This indicated that such a coupling method appears particularly well suited for SMFs and PCFs connections.

Early in 2008, a technique for fabricating micro- and nano-sized self-written waveguides induced in a range of free-radical photo-polymerization systems was presented by Soppera and co-workers [12,116]. In a photo-polymerizable formulation containing a triacrylic monomer base (pentaerythritol triacrylate), a Xanthenic dye sensitizer (Eosin Y), and a co-initiator (methyldiethanolamine), polymer micro-objects were produced by a 532 nm laser diode. Another free-radical photo-polymerizable system was then studied, involving three main components, i.e., a Xanthenic dye sensitizer, a co-initiator (methyldiethanolamine, 8 wt %), and a liquid multifunctional monomer base, which is the solvent of the other products. SWW growth was observed for different dye concentrations, including the effects of the surrounding atmosphere. A novel method for fabricating polymer waveguides between two optical fibers, using the self-guiding effect, was introduced. The aim was to connect light from two counter propagating optical fibers whose extremities are co-aligned and linked together by a drop of liquid photo-polymerizable formulation. By doing so, two polymer tips can easily be grown from each of the fiber ends.

Later in 2008, Acrylamide/Polyvinyl Alcohol (AA/PVA) photopolymer solutions, i.e., water based liquid solutions, were shown to exhibit self-focusing and self-trapping behaviors, by Jisha et al. [11]. As previously presented in Section 1.2 extensive studies examining the use of AA/PVA photopolymer layers for use in producing volume holographic gratings have been reported [23,66,68,75,89,108,109,110,134,135,136,137,138,139]. In all of these cases, the AA/PVA photopolymer was prepared as a dry thin film. In [11], it was demonstrated that self-written waveguides can be formed in liquid AA/PVA in a cuvette. However, it is notable that the resulting self-written waveguides formed in such liquid photopolymer systems were not stable over time but dissolve, or precipitate completely out post-exposure. Therefore, in order to study self-writing and self-focusing it is necessary to determine if it is possible to record permanent self-written waveguides in a dry AA/PVA volume.

Followed previous experimental work reported in [12,116], Jrad et al. (i.e., Soppera and co-workers) [117] further improved the production of polymer micro-lenses at the ends of optical fibers. A method to tailor the geometry of the self-written polymer-tips by adjusting the physico-chemical parameters to obtain the expected light-induced polymerization process, was presented. Numerous physico-chemical and photonic parameters were examined which provided a better knowledge of the polymer micro-component building up. Which allowed key parameters to be specified and their contributions to the final shape of the tip to be determined. In this way, the manufacturing technique was shown to be fast, highly flexible (curvature radii ranges from 0.2 to 200 μm) and was demonstrated not to require expensive equipment.

In the meantime, Soppera and co-workers [13] demonstrated that micro-pillars and micro-lenses can be integrated at the end of optical fibers. The micro-fabrication was carried out by direct photo-polymerization in the near-IR region (700–950 nm). A self-guiding polymerization system was generated using the NIR-light emerging from the optical fiber. The photopolymer material used in this work consisted of a mixture of three components, i.e., a sensitized dye (5,5′-dichloro-11-diphenylamino-3,3′-diethyl-10,12-ethylenethiatricarbocyanine perchlorate), a co-initiator (4-aminobenzoic acid ethyl ester from Sigma-Aldrich, St. Louis, MO, USA), and a triacrylic monomer (pentaerythritoltriacrylate from Sartomer). The experimental results demonstrated that free-radical photopolymer materials can be used to fabricate micro-optical devices in a single step non-contact manufacturing process utilizing NIR activation.

Pang et al. [140] proposed and demonstrated that a polymer-tipped optical fiber can efficiently couple light between a submicron size self-written waveguide and a single-mode fiber. Compared to the performance of a commercial micro-lens, for a self-written waveguide with a taper (2 × 0.3 μm^2^) and a ROC (1.55 μm), the coupling efficiency was enhanced by a factor of 1.3. Experimental results were observed to be in agreement with numerical simulations confirming the evanescent nature of the coupling taking place in the case of such untappered waveguides. Thus, the technique introduced in this work appears to be an excellent candidate to couple light into sub-micrometer self-written waveguides.

Later in 2009, for the first time, a numerical model which showed the possibility generating self-trapping beams in a photopolymer material i.e., phenanthrenequinone doped poly(methyl methacrylate) (PQ/PMMA), was developed by Kashin et al. [123]. In this model the diffusion theory in PQ/PMMA photopolymer material was investigated and a diffusion material model for calculating the related refractive index change was derived. This theory was further combined with the physical paraxial wave model. The resulting paraxial scalar wave equation was solved by employing the Split-step Fourier Method in Fourier space, and then combined these results with regular updates of the refractive index which can be obtained from the diffusion theoretical model. This model enabled a more accurate and physical description of the evolution of self-written waveguides optically induced in photopolymers.

The results of the numerical simulation of self-trapping in PQ/PMMA photopolymer performed by [123], were confirmed when corresponding experimental formations of a straight waveguides channel structures (as a result of the predicted self-action) were demonstrated by Tolstik et al. [14,15]. The experimental validations were carried out in two different geometries of the PQ-containing materials, i.e., in planar layer [14] and bulk sample [15], respectively.

In the planar geometry relatively thin PQ/PMMA layers were prepared that were several hundreds of microns thick [14]. Experiments were carried out for several identical PQ/PMMA material samples having layer thicknesses of 400 μm and a PQ-concentration of 2.5–3.0 mol %. An Ar-laser source (514.5 nm) was used and the light power required to form a channel was found experimentally to be in the range of milliwatts. It was found that the formation of the waveguide was strongly influenced by the heating of the material by light during exposure leading to an additional thermal defocusing within the material of the light beam. Therefore, the numerical model of the description of the laser beam propagation in PQ/PMMA was improved by considering the heat conduction equation. The formation of a self-trapped channel with a length of 6 mm was observed by applying Ar-laser illumination for relatively long exposure times of 5–6 min. The predictions were in good agreement with the experimental results.

In 2014, bulk PQ/PMMA samples were prepared having dimensions of millimeters [15]. Their shape was determined by using a glass mask (usually the size of samples is 20 × 30 mm^2^ with a thickness of 3–5 mm). Self-channeling was always achieved in such bulk PQ/PMMA media with thicknesses up to several millimeters and 0.1 mol % PQ-concentration. Waveguide structures were generated with a length of 2–3 cm at different laser wavelengths (405 nm, 488 nm, and 514.5 nm). A theoretical model was developed which included two competing waveguide formation mechanisms, namely the process of photo-attachment of PQ molecules with the formation of a photoproduct (positive change of the refractive index Δ*n*_PQ_) producing focusing and the negative change of the refractive index (Δ*n*_T_) by heating of the medium producing spreading. The predictions of this theoretical model were shown to be in good agreement with the experimental results.

Missinne et al. [118] demonstrated that a low-loss self-written waveguide can be fabricated in photopolymers to interconnect two single mode telecom fibers. Modeling and optimization of the curing kinetics for either a single material (Acrylate, NOA-68), or two-material (two types of Ormocer) system were carried out experimentally and theoretically. In the experiments, organically modified ceramic and acrylate materials were studied, which are commercially available materials. The Ormocer materials [118] have low absorption losses (around 0.5 dB at 1550 nm), ease of use and high stability, which are widely used for optical interconnects. NOA-68 is sold as an optically clear UV curable adhesive, as well as used for holographic recording. In theoretical analysis, this single material system was examined because of its use in industrial applications. The two-material approach was introduced, i.e., simplifying the study of the polymer waveguide medium, therefore the final optical insertion loss of the fiber-waveguide-fiber structure. Both approaches enabled easy process monitoring and optimization, resulting in total insertion losses below 0.3 dB for SMF–SWW–SMF transition at 1550 nm.

In 2015, Belgacem et al. [141] demonstrated that the diffusion of the monomer has an important influence on the interaction between LISW waveguides. This is especially important when writing optical components like Y or X junctions, or LISW couplers. A radical photopolymerizable formulation compose of tri-functional acrylate monomer, the pentaerythritoltriacrylate (PETA) monomer (~90 wt %), a Xanthenic dye sensitizer Eosin Y (~0.5 wt %), and a co-initiator methyldiethanolamine (MDEA) (~9.5 wt %) was used. The addition of a chemical in order to implement specific optical functions: e.g., electro-optical, fluorescence or photochromic, may also plasticize the material and therefore enhance diffusion in the material. Such effects have to be considered when fabricating LISW channel based devices. A numerical model was developed by Belgacem et al. for the material that represents a good trade-off between the simplicity required for computation and the complexity of a full complete photopolymerization model. The numerical results using this model allow a better understanding of the construction of LISW optical waveguides, and allow also suggesting several approaches to control and optimize the self-written waveguides.

In 2016, Burrell et al. [142] reported a study about Polymer Waveguides (PWGs) which are typically aligned to silica-based optical fibers for coupling. PWGs are used within photonic interconnects as cheap and versatile substitutes for traditional optical fibers. In this work an epoxide elastomer is applied and cured at the interfaces to achieve index matching and rigid attachment. It has been proposed to use the SWW technique as an alternative to further reduce connection or Insertion Loss (IL) and alleviate marginal misalignment issues. We note that the elastomer material was deposited after the initial alignment. SWWs are formed in the material using ultraviolet light (UV) as an exposing beam. The coupling of UV light cures a channel of material between the two differing structures or components, was formed. There are several factors that effect on the resulting SWWs, such as longitudinal gap distance, UV cure time, input power level, polymer material selection and choice of solvent. Experimental results of purely index-matched samples are compared with SWWs at the fiber-PWG interface. This shows that <1 dB IL per connection can be achieved by either method and the results indicate that the lowest potential losses which can be achieved are associated with a fine-tuned self-writing process. Through this work, we strongly concluded that using the SWWs is useful to reduce an overall processing time and enable an effectively continuous low-loss rigid interconnect.

## 5. Conclusions

In summary, it is clear that an extensive amount of work has been carried out to investigate the hologram and self-writing technique applied in photopolymer materials in recent years. Our paper involves a brief review of theoretical models and detailed material characterization to optimize material compositions and performances.

We begin in this paper by introducing a brief review of photopolymer materials and discussing the fundamentals of holography. The review of the theory of optical diffraction by both thin and thick (volume) gratings was also presented. The recording of unslanted transmission type volume holograms was discussed. It was emphasized that our analysis is focused on gratings recorded in the Acrylamide/Polyvinyl Alcohol (AA/PVA) photopolymer. Following this, Kogelnik’s first-order two-harmonic coupled wave theory, which relates holographic diffraction efficiency to material refractive index modulation, is briefly reviewed. Then, the history of photopolymers for Holographic Data Storage (HDS), with an emphasis on AA/PVA materials, is reviewed. A description of material preparation and the composition of the AA/PVA photopolymer layers used to produce holographic gratings, are given.

Finally, another important optical application of such free radical photopolymer materials, i.e., self-written waveguides, is introduced. First, a brief review of the self-writing process is discussed in detail in the self-developed materials. Then, some previous studies and the related applications of the photopolymers for the introduced Light Self-writing Technique (LST) are discussed.

## Figures and Tables

**Table 1:** Components of the photopolymer material used in this study.

Component	Function	Per 100 cm^3^
Polyvinyl Alcohol (PVA)	Binder	70 cm^3^ of 10% sol.
Acrlyamide (AA)	Monomer	2.4 g
Bis-acrylamide (BA)	Cross-linker	0.8 g
Xanthene dye	Dye	16 cm^3^ of 1.25 × 10^−^^3^ M
Triethanolamine (TEA)	Electron Donor (ED)	8 cm^3^
